# Simultaneous Right Retroperitoneal Schwannoma and Left Renal Hydatid Cyst

**DOI:** 10.1155/2013/467192

**Published:** 2013-07-11

**Authors:** Ali Kamalati, Hamid Tabrizchi

**Affiliations:** ^1^Department of Urology, Shahid Bahonar Hospital, Kerman University of Medical Sciences, Kerman 7613614570, Iran; ^2^Department of Pathology, Kerman University of Medical Sciences, Kerman, Iran

## Abstract

Retroperitoneal schwannomas are quite rare tumors. Isolated renal hydatid cyst is also rare, and it forms 2–4% of hydatid disease. Because of their infrequent occurrence, nonspecific signs and symptoms, and lack of distinguishing radiologic features, we report herein a case of right retroperitoneal mass in a 26-year-old woman which was found to be benign schwannoma following a percutaneous core needle biopsy and a large cortical cyst in the lower pole of the left kidney which was diagnosed as isolated renal hydatid cyst following exploration.

## 1. Introduction

Schwannomas originate from Schwann cells of peripheral nerve sheaths, and while they are commonly seen in head, neck, and flexor surfaces of the extremities, they are quite rare in the retroperitoneal region. Schwannomas are most often diagnosed by histologic examination and immunohistochemical staining of the excised mass.

Hydatid disease is caused by the larval stage of *Echinococcus granulosus,* in which humans are an intermediate host. Renal hydatid cysts are also rare and correspond to 2–4% of all cases of hydatid disease.

Here we report an incidentally diagnosed bilateral retroperitoneal masses in a young woman which were found to be right benign schwannoma following a percutaneous core needle biopsy and left renal hydatid cyst following exploration. The therapeutic approach is also presented.

## 2. Case Report

**Figure 1:**
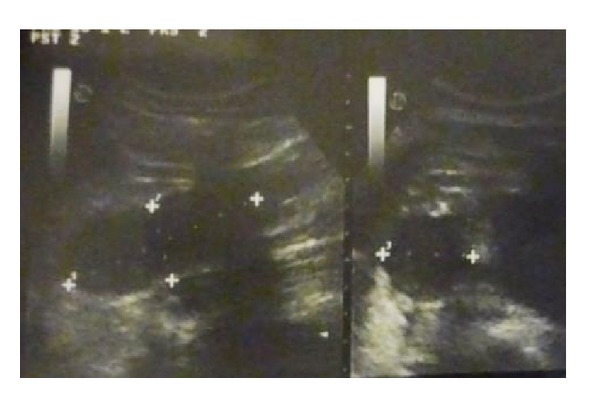
Sonographic images revealed a well-defined mass with lobulation and hypoechoic content in the posteroinferior of the right kidney.

The present case is a 26-year-old woman. One year prior to the current admission, in an ultrasonography study, a well-defined lobular mass (78 × 28 × 33 mm in size) with a hypoechoic content in the posteroinferior of right kidney and a univesicular cyst of 105 × 98 × 73 mm in the lower pole of left kidney causing mild hydronephrosis had been reported (Figure 1). The internal structure of the left mass was anechoic, and the cystic wall was thin and relatively regular. No alteration in the wall's structure, which could resemble a germinative membrane, was apparent.

**Figure 2:**
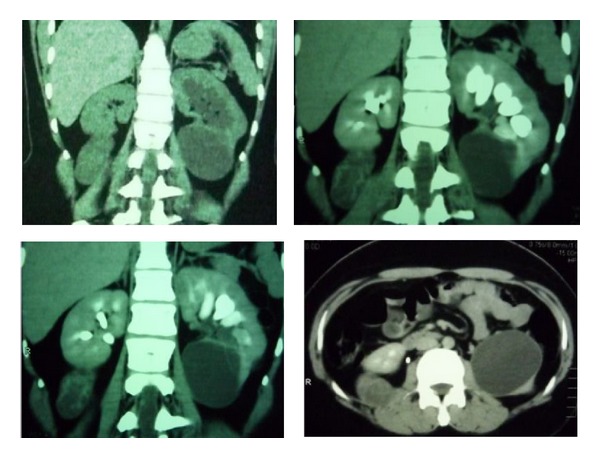
CT images demonstrate a retroperitoneal mass on the right side with heterogenous contrast enhancement and large cortical cyst in the lower pole of the left kidney with thin peripheral contrast enhancement without any other organ involvement.

In noncontrast CT-scan, the right mass had been hypodense and it was heterogeneously enhanced following contrast injection and a large cortical cyst in the lower pole of the left kidney with thin peripheral contrast enhancement without any other organ involvement (Figure 2).

There had been no urinary symptom, gross hematuria, headache, sweating, flushing, or tachycardia. Physical exam had been normal, and abdominal masses had not been palpable.

Her medical history was also unremarkable, and no family history of hydatid disease was identified.

Medical laboratory tests including CBC, urinalysis, urine culture, and blood chemistries had been all in normal range. The patient had not been referred due to the lack of any symptom until feeling occasional mild and vague pain in her right flank from 2 to 3 months prior to the current admission.

**Figure 3:**
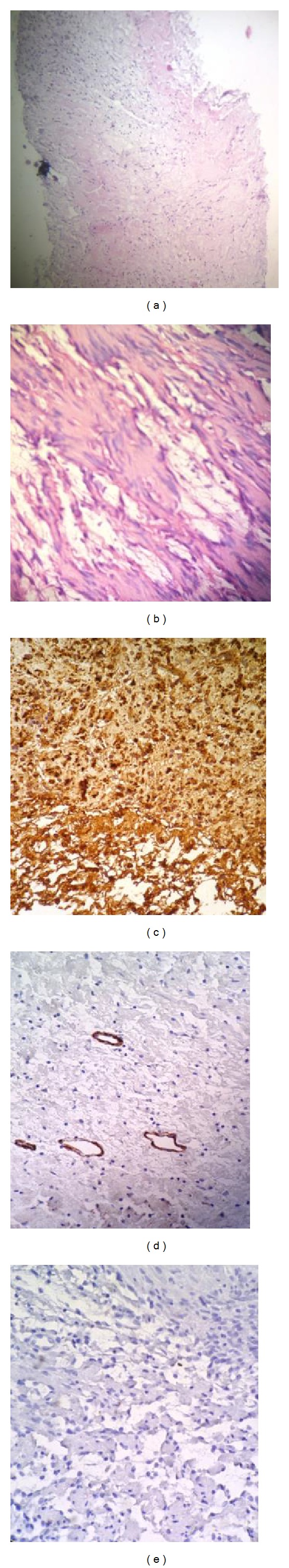
(a) and (b) H&E stained sections: (a) low power view of core needle biopsy: Antoni-A and -B growth pattern ×25, (b) palisading arrangement of spindle shape tumor cells (Verocay bodies) ×100; (c) I.H.C study with S-100 antibody shows positive nuclear reaction (PAP staining ×40); (d) and (e) I.H.C studies with SMA and CD 117 both show negative reaction with tumor cells (PAP staining, D ×40 and E ×100).

**Figure 4:**
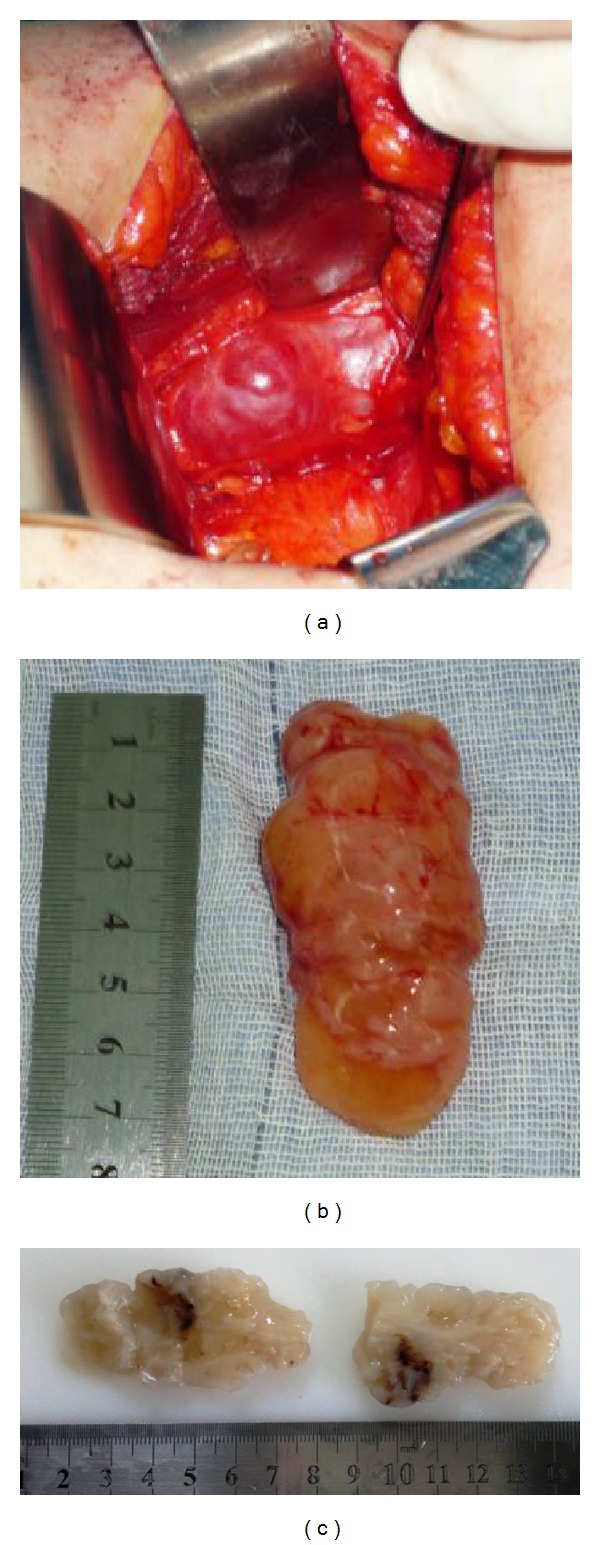
The right retroperitoneal mass (a), measured 7 × 3 × 3 cm, was removed (b). Hemorrhagic, cystic, and myxoid changes are seen through a cut section from the mass (c).

In the second admission, no significant change in the masses was observed and all medical laboratory tests were normal. Histologic study following ultrasound-guided percutaneous core needle biopsy of the right retroperitoneal mass revealed benign schwannoma (Figure 3). Open surgery with right subcostal flank approach was performed to remove the mass (Figure 4(a)). A well-encapsulated tumor in the right retroperitoneal region and anterior part of psoas muscle next to the inferior pole of the right kidney without any adhesion was observed. The tumor was enucleated and excised easily (Figures 4(b) and 4(c)). There was no intraoperative lymphadenopathy or bleeding.

Radiologic findings led us to drain the cyst percutaneously with the diagnosis of left simple renal cyst.

After three month, using local anesthesia, the cyst was punctured with an 18-gauge Chiba needle under ultrasound guidance; 120 mL clear fluid was aspirated. Then 50 mL of 95% ethanol (40% of the cyst volume) were injected into the cavity. The ethanol was left for 20 min following which all ethanol was aspirated and the needle was withdrawn. Biochemical and cytological analysis of the cyst was unremarkable and culture was negative.

No evidence of retroperitoneal tumor recurrence and no significant change in the left renal cyst were observed in the ultrasound followup performed three months later.

Because the left mass was diagnosed as a large simple renal cyst, an operation with flank position was planned. At exploration, the large cystic mass in the lower pole of the left kidney presented with a regular but thick wall. Aspiration of the cystic fluid was down. It was like clear water. When the cystic wall was opened, sloughed and wrinkled daughter cysts were seen. After taking precaution to prevent hydatid seeding, by means of sponges saturated with povidone iodine, the daughter cysts were extracted. The internal surface of the cyst wall was washed with povidone iodine, and cystectomy was performed, leaving a portion of the cyst wall at the renal side.

Followup at 6 months and 1 year with ultrasonography revealed no evidence of tumor recurrence or hydatid disease in the perinephric space from spillage of the hydatid fluid.

## 3. Discussion

Schwannomas (neurilemmomas) are neurogenic benign tumors arising from schwann cells of peripheral nerve sheaths. While schwannomas commonly appear in head, neck, and extremities, they are quite rare in the retroperitoneal region, so that just 0.7% of benign and 1.7% of malignant types are observed in this region [1–3]. Schwannomas have a slow and prolonged clinical course before the diagnosis. Malignant transformations are uncommon in these types of tumors. Histologically, they are encapsulated and show various areas from dense cellularity of spindle-shaped cells called Antoni-A (AA) regions to hypocellular areas of myxoid matrix called Antoni-B (AB) regions (Figure 3(a)) [4].

Immunohistochemical (I.H.C) staining is typically positive for S-100 (Figure 3(c)), vimentin, and neuron-specific enolase and is negative for smooth muscle action (SMA) and CD117 (Figures 3(d) and 3(e)).

Preoperative diagnosis is often difficult. In facing retroperitoneal tumors, schwannomas should be in mind. Schwannoma manifests as a well-defined homogenous mass in CT-scan, and following intravenous contrast injection its fibrous capsule has rim enhancement [5].

Nowadays mass needle biopsy has been reconsidered and paid more attention particularly in young patients and those who are potential candidates of different therapeutic approaches from observation to surgical excision.

Core needle biopsy is simple and safe and has the accuracy rate of 98% in differentiating soft-tissue benign tumors from malignant ones [6].

Management options are ranged from radiologic followup in asymptomatic patients to surgical resection in symptomatic patients. Subtotal resection in order to minimize the risk of surgery and preserve adjacent vital tissues in benign types is recommended. benign schwannomas have good prognosis, and recurrence is very rare [7].

Hydatid disease is a worldwide zoonosis caused by the larval stage of the cestode *Echinococcus spp.* (*E. granulosus*, *E. vogeli*, and *E. multilocularis*). Human infection is most common in sheep-herding areas, such as Australia, Argentina, Spain, Greece, and the Middle East. Dog is the principal definitive host, and sheep is the most common intermediate host. Human may become intermediate host through contact with a definitive host or ingestion of contaminated water or vegetables. Virtually all parts of the human anatomy have been reported to have hydatid cysts [8]. The cysts are located in liver (75%), lungs (15%), and other organs (10%). Renal involvement occur in only about 2–4% of cases, and cysts are most frequently located in the lower pole of the kidney [9–12].

There are no specific symptoms or signs that will reliably confirm the diagnosis of renal hydatid disease. The combination of clinical history, radiologic findings, and serological and urinary studies yields a reliable diagnosis in only 50% and a presumptive diagnosis in 71% of cases [13]. Renal hydatid cyst may remain silent for years or may be complicated with infection, abscess, necrosis, hemorrhage, or obstruction [12]. The most common symptoms are dull flank pain and mass. 

The cyst may rupture into the collecting system, and the patient experiences severe renal colic and passage of debris resembling grape skins in the urine (hydatiduria), which is pathognomonic and seen in 5–25% of all renal hydatidosis [13].

Ameur and coworkers reported 34 cases with renal hydatid cysts. Clinical presentations were pain (63%), hematuria (31.4%), mass (26%), hydatiduria (11.4%), prolonged fever (23%), and hypertension (3%), respectively [14]. Also, there are no pathognomonic serological or immunological tests for hydatid cyst [12].

 Radiological studies have an important role in the diagnosis of renal hydatid disease. Intravenous pyelography (IVP) typically shows a thick-walled cystic mass, occasionally calcified.

Ultrasonography usually shows a multiloculated or multicystic mass.

On CT scan, several patterns of renal hydatidosis may be presented. The most specific pattern is a cystic mass with discrete and round daughter cysts and a well-defined enhancing membrane. The less specific is that of a thick-walled multiloculated cystic mass. The presence of daughter cysts is strongly suggestive of hydatid disease and differentiates the lesion from a simple renal cyst, renal abscess, infected cysts, and necrotic neoplasm.

 Nevertheless, radiologic findings are usually suggestive but inconclusive in diagnosis of renal hydatid cyst [13]. Parenchymal saving surgery remains the mainstay of treatment of renal hydatid cyst [15]. It may range from open surgery to laparoscopy and least invasive PAIR (percutaneous aspiration injection and respiration) technique [16].

## 4. Conclusions

1. Retroperitoneal mass needle biopsy is simple, safe, and accurate in differentiating soft-tissue benign tumors from malignant ones and has been paid more attention.
2. Hydatid cystic masses may not always present with characteristic radiological findings, and extreme caution should be practiced by the surgeon and radiologist in order to prevent iatrogenic echinococcal dissemination.
